# The right to be informed and fear of disclosure: sustainability of a full error disclosure policy at an Italian cancer centre/clinic

**DOI:** 10.1186/s12913-015-0794-3

**Published:** 2015-04-01

**Authors:** Stefano D’Errico, Sara Pennelli, Antonio Prospero Colasurdo, Paola Frati, Lorella Sicuro, Vittorio Fineschi

**Affiliations:** ASL2 Lucca, Ospedale ‘Campo di Marte’, edificio O, 55100 Lucca, Italy; I.R.C.C.S. Centro di Riferimento Oncologico della Basilicata, via Padre Pio 1, 85028 Rionero in Vulture (PZ), Italy; Department of Anatomical, Histological, Forensic and Orthopaedic Sciences, Sapienza University of Rome, Viale Regina Elena 336, 00185 Rome, Italy; National Institute of Statistics ISTAT, AEM Territorial Office for Abruzzo and Molise Regions, Pescara, Italy

**Keywords:** Medical errors, Information, Full disclosure policy, Clinical risk management

## Abstract

**Background:**

The aim of this study was to investigate the behaviour of physicians in cases of medical error as well as the nature of the information that should be given to the patient and to ascertain whether it is possible to institute a full error disclosure policy. Data was collected through the completion of anonymous questionnaires by medical directors of the IRCCS CROB (the Oncology Centre of Basilicata, Italy).

**Methods:**

An anonymous questionnaire consisting of 15 questions was prepared and administered to all the physicians working at the IRCCS CROB – the Oncology Centre of Basilicata. The main aim of the research was to evaluate the feasibility of adopting a full disclosure policy and the extent to which such a policy could help reduce administration and legal costs.

**Results:**

The physicians interviewed unanimously recognize the importance of error disclosure, given that they themselves would want to be informed if they were the patients. However, 50% have never disclosed a medical error to their patients. Fear of losing the patient’s trust (33%) and fear of lawsuits (31%) are the main obstacles to error disclosure.

**Conclusions:**

The authors found that physicians were in favour of a full policy disclosure at the IRCCS CROB – the Oncology Centre of Basilicata. Many more studies need to be carried out in order to comprehend the economic impact of a full error disclosure policy.

**Electronic supplementary material:**

The online version of this article (doi:10.1186/s12913-015-0794-3) contains supplementary material, which is available to authorized users.

## Background

Since the early 90s there has been a growing awareness that medical treatment may cause harm to patients. Numerous studies cited in international scientific literature report that the percentage of adverse cases amongst hospital patients is between 0.4% and 16%. Data provided by the U.S. Institute of Medicine (IOM), showing similar findings, estimates that the death toll from avoidable medical errors occurring in US hospitals is between 44,000 and 98,000 [1]. According to the World Health Organization (WHO), a medical accident occurs when a patient experiences an event which could or does cause injury [2].

Over 43% of patients claim to have experienced an ‘adverse event’, mainly as a result of the wrong therapy, nursing error, problems with the medical team or the wrong diagnosis [3,4]. The principal factors causing ‘adverse events’ or errors are said to be: the scarce amount of time at the disposal of health practitioners for their patients, work overload, stress and tiredness experienced by the medical staff, miscommunication between members of the medical team and shortage of personnel. When an error occurs it has a physically, emotionally and economically traumatizing effect upon the patient. Patients suffer a range of negative emotions including sadness, anxiety and depression. In addition, patients are angered by the fact that the error was predictable and could have been prevented, and they fear that further errors or adverse events might occur [5]. In any case, documented research shows that the patients and their families expect those responsible to acknowledge their error and give a reason for the adverse event. It is well known that iatrogenic injuries are accompanied by a high degree of tension in the patient-doctor relationship. Patients want information about errors which can occur during treatment. Moreover, it is their right to receive such information. At the same time, the medical staff should consider it their moral and ethical duty to acknowledge errors and discuss them with the patient and family.

The practice of error disclosure was first proposed by the American Medical Association in 1982. Some years later in 2001 the Joint Commission endorsed error disclosure and envisaged its implementation as an instrument of clinical risk management to help reduce lawsuits to seek redress [6,7].

Recent international scientific research focuses a great deal of attention on the circumstances of error disclosure to the patient, on the reactions of the patient and family members at the time the error occurs and on the nature of the information that should be given to the patient [8-14]. Although it emerges from research that physicians are generally willing to admit their mistakes to patients and family, this does not always happen in practice because of the widely diverging and incompatible priorities of the parties involved [15-20]. Kaljian LC et al. show that although most physicians recognize the ethical and deontological importance of error disclosure, (93% being favourable in the case of serious injury and 97% in the case of mild injury), in practice only 41% of the physicians interviewed had actually admitted responsibility for a medical error resulting in mild injury to the patient, and only 5% in cases of serious injury [21].

Very little data is available for Italy regarding this problem. The only relevant study carried out in Italy by Vincent JL in an Intensive Therapy Unit, and published in a European review, shows that only 11% of physicians declared that they provided their patients with complete information (even when an error occurred), whereas 74% usually limited the information they provided to a mitigated version of the facts even though they recognized a moral and deontological duty to give complete information [22].

The aim of this study is to investigate the behaviour adopted by physicians in cases of error and to ascertain whether it is possible to institute a full error disclosure policy. Data was collected through the completion of anonymous questionnaires by medical directors of the IRCCS CROB (the Oncology Centre of Basilicata). The survey of 15 questions was compiled after extensive research and is divided into 3 sections. The first refers to the personal data of the physician completing the form, the second contains specific questions on the type of guidance in behavioural strategy given to health care providers in the event of mistakes, and the third section focuses on the nature of the information about the error and how it is communicated to the patient and family.

## Methods

### Study setting and data collection

On 2012, September the 20th, this study was approved by the IRCCS CROB Ethical Committee. All procedures performed in this study were in accordance with the ethical standards of the institution or practice at which the studies were conducted. From October to November 2012 a cross sectional survey was administered to medical directors working at the IRCCS CROB in Rionero inVulture.

The questionnaires were distributed directly to managerial members of the medical staff in the medical department, in surgery and in the service operating units of the IRCCS CROB (Additional file 1). Once completed, the anonymous surveys were returned to the Quality and Risk Management Department of the Central Administration where the data was processed.

An ethical review by local/institutional committee was not required for this type of study.

### Analysis and data

Descriptive and inferential statistical data was analysed. In particular, both the frequency and percentage of each type of reply for the different categories were calculated. The average and standard deviation was calculated for the numerical variables of age and years of service. A bivariate evaluation was then carried out in order to analyse the relationship between pairs of variables. The Fisher test was especially useful for investigating significant statistical differences between categorical variables; a p-value of less than 0.05 indicated statistical significance.

## Results

### Demographic aspects

48 surveys were collected from the following areas: 50% were completed by members of the medical staff (24 respondents in total); 31% were surgeons (15 in total), and 19% were the diagnostic staff (9 in total). 67% of the surveys were completed by males (32 in total), and the remaining 33% by females (16 in total) (Table 1).

**Table 1:** **Demographic data relating to survey participants**

**Area**	**Medical**	**Surgical**	**Diagnostic**	**Total**
Average age (SD)	44 (9)	42 (7)	45 (19)	44 (8)
Gender				
M	14 (58%)	12 (80%)	6 (67%)	32 (67%)
F	10 (42%)	3 (20%)	3 (33%)	16 (33%)
Average number of years of service (SD)	14 (9)	11 (6)	15 (9)	13 (8)

38% (18 respondents in total) of the medical staff claimed to have made mistakes in the last year of work, while 54% (26 in total) claimed that they had not made any mistakes, and the remaining 8% did not reply to the question. The respondents who admitted to having made a mistake over the last year work in the following fields: 39% (7 in total) work in surgery, 28% (5 in total) in diagnosis and 33% (6 in total) in medicine.

### Physicians’ inclination to error disclosure

In reply to the question: “Have you ever disclosed a medical error to a patient?”, 50% of the medical staff said they had never disclosed an error to a patient, 22% said they had but only because the patients themselves had asked them to explain what happened, while 11% and 17% stated that they disclosed errors in cases of mild injury and serious injury respectively.

An analysis of the answers to the question: “Do you think that patients should be informed if a mistake is made because if you were in their situation you would expect to be informed?” reveals that 100% of the subjects questioned answered affirmatively.

Another significant finding is that 78% of the physicians who had made a mistake felt satisfied after having discussed it with the patient.

Reluctance to adopt a full error disclosure policy is motivated primarily by the fear of losing the trust of the patients (33%; 6 in total) and fear of legal claims for redress (31%; 15 in total).

52% (25 respondents in total) of the physicians interviewed believe that the doctor who made the mistake should be the one to inform the patient, while 35% (17 in total) consider it essential that members of the management team, and especially the medical director, are present at the meeting with patient and family. There was unanimous agreement that physicians should be supported by the clinical centre managing system when an error occurs. In fact, all the medical staff gave an affirmative response to the following question: “In the case of an error, do you think that the clinician responsible has the right to adequate psychological and emotional support from the clinical centre managing system?” Demographic factors of age, sex and medical profession have no significant influence on the physician’s propensity to disclose error.

### How error disclosure is perceived

87% (42 respondents in total) of physicians believe that it is an ethical and deontological duty to admit to the patient that a mistake has been made. On the other hand, 54.2% (26 in total) of the respondents believe that it is the patient’s right to be informed of errors occurring during the course of their medical treatment (Table 2). Out of 23 physicians responding to the question: “Do you think it is necessary to reveal mistakes to patients every time they happen?” 18 in total (78%) answered affirmatively, while 5 in total (22%) said “no”. A statistically significant difference (p = 0.016) from what the results appear to indicate was observed. 18 physicians (37% of the respondents) who declared that they had never made a mistake, nevertheless agreed that, for patients, the most important input at the time of error disclosure is reassurance that the problem will be resolved rapidly.

**Table 2:** **Perception of error disclosure, relative frequency and percentage of replies returned**

**Questions**	**Yes**	**No**	**Missing**
**Do you think that admitting a mistake to the patient is:**			
wrong	1 (2,1%)	13 (27.1%)	34 (70.8%)
an ethical and deontological duty	42 (87.5%)	0 (0.0%)	6 (15.5%)
the patient’s right	26 (54.2%)	1 (2.1%)	21 (43.8%)
necessary every time a mistake happens	18 (37.5%)	5 (10.4%)	25 (52.1%))
only necessary in cases of serious injury	4 (8.3%)	15 (31.2%)	33 (68.8%)
only necessary in cases of mild injury	1 (2.1%)	13 (27.1%)	34 (70.8%)
only necessary in cases where the patient is not harmed	2 (4.2%)	12 (25.0%)	34 (70.8%)
only necessary when the patient asks for an explanation	2 (4.2%)	12 (25.0%)	34 (70.8%)
helpful in avoiding medical lawsuits?	14 (29.2%)	4 (8.3%)	30 (62.5%)
**Do you think that admitting a mistake to the patient:**			
strengthens the patient’s trust in the doctor	31 (64.6%)	6 (12.5%)	11 (22.9%)
reduces the risk of medical lawsuits	23 (47.9%)	6 (12.5%)	19 (39.6%)
reduces the probability of the same error being repeated	23 (47.9%)	4 (8.3%)	21 (43.8%)
reduces the patient’s apprehensions about the likely outcome	12 (25.0%)	8 (16.7%)	28 (58.3%)
reduces the probability that the patient will change doctors and/or hospital?	14 (29.2%)	7 (14.6%)	27 (56.2%)

### Training on how to approach error disclosure

Most of the medical directors interviewed (52%; 25 in number) do not think specific training is required in order to guarantee a correct approach to error disclosure. Nevertheless, 83% (4 in number) consider ad hoc training courses to be useful. The Fischer test did not produce any statistically significant difference between physicians’ answers to the following two questions: “Have you ever disclosed a medical error to your patients?” and “Do you think an ad hoc training course on how to approach error disclosure is necessary and useful?”.

## Discussion

The results of this study show that among the medical directors interviewed, there is unanimous recognition of the importance of error disclosure, given that they themselves would want to be informed if they were the patients. However, 50% have never disclosed a medical error to their patients. There is a discrepancy between the frequency of error disclosure in cases of serious injury (17%) and in cases of mild injury (11%).

This evident, and at the same time predictable contrast between theory and practice confirms the finding of studies in the international scientific literature [23]. The findings of a major study in the field by Ghalandarpoorattar et al. confirm that although physicians express willingness to disclose errors to patients quite frequently, they do not put their intentions into practice. From the same study it emerges that the tendency to disclose errors causing serious injury is greater (49.1%) than when the injury is slight (39.6%) [24].

Certainly, in every country, error disclosure to the patient is affected by limitations of an emotional, cultural and attitudinal nature. There is a fear of revealing the error because of the consequences that might ensue. This reinforces the idea that disclosure is dangerous as well as useless. Finally, the practice of error disclosure is entirely alien to cultural norms and medical studies [25].

Recently, the Italian code of medical ethics introduced a new dimension to the issue of supplying medical information to the patient. The doctor has an ethical duty to inform the patient of each unwanted event and its causes, and to identify and report adverse events, near misses and procedural and diagnostic errors [26]. Article 14 states: “The doctor will work to ensure the most appropriate conditions of patient safety as well as the adequacy of medical facilities and the prevention and management of clinical risk, through:

- adherence to good clinical practice;
- attention to the process of giving information and obtaining consent, and to the reporting of an adverse event and its causes;
- the continued development of training and evaluation with regard to safe care procedures;
- the detection, reporting and evaluation of sentinel events, errors, ‘near-misses’ and adverse events, ascertaining the causes and ensuring confidentiality about the information collected’ [27].

The answers given by physicians interviewed in our study show that the main obstacles to error disclosure are: fear of losing the patient’s trust (33%) and fear of lawsuits (31%). These findings correspond with the results of the study carried out by Ghalandarpoorattar et al. in which the same two factors (fear of losing the patient’s trust and fear of lawsuits) were found to be the main reasons discouraging physicians from disclosing errors to patients. Worthy of reflection are the findings of Gallagher et al. which show that 60% of the physicians interviewed disagree with error disclosure to patients, fearing that the patients would not be able to grasp the information given [28].

At an international level, there is a general consensus among bioethicists, physicians and organizations representing patients, that it is the ethical and deontological duty of physicians to inform patients when harmful medical errors occur.

This was also found to be the case in 87.5% of the survey replies given by professional health care providers who consider it to be, first and foremost, an ethical and deontological duty to disclose medical errors to patients.

The well-known principle *primum non nocere (first do no harm)* has always inspired doctors and health care providers, who have a legal duty to safeguard the health of citizens. All professional health practitioners are ethically and morally bound to observe the principle of error disclosure in respect of the constitutional rights of citizens.

In other words, we need to reconcile the rights of patients to be informed so that they may decide for themselves, with the obligations of the physician to provide information. Furthermore, information should be provided not merely for the purpose of obtaining the patient’s free and willing consent to treatment, but in order to secure his or her full involvement in the various processes of that treatment and its unforeseeable results.

The occurrence of an undesirable event is certain to provoke tension between patient and health care provider. However, the need, so often expressed by citizens, to have an adequate explanation of the technical and diagnostic procedures pertaining to the error, cannot be ignored.

An analysis of the results shows that physicians consider that, on disclosure of the mistake, adequate information should be given to reassure the patient that everything possible will be done to correct the error (60%) and that steps will be taken to prevent its repetition (48%). The very same physicians who have never disclosed an error to patients are the ones who believe that error disclosure should be accompanied by reassurance that everything is being done to repair injury caused to the patient.

The analysis carried out by Gallagher et al. produced similar results to the findings proposed above [28]. Based on patient feedback, the study provides guidelines as to the nature of the information that should be given in the event of error. The information must be clear and complete: i.e. the reason for the error, the consequent effect upon the patient’s health and information on what needs to be done to repair injury.

It emerges from the study that the patient expects the physician and the health centre to admit that they have made a mistake and to learn from their error by taking all the necessary steps to prevent its recurrence in the future. The physicians interviewed also stated that in cases of error, assistance given by the Health and General Administration of the hospital in communicating the mistake to the patient is absolutely vital, as it proves that the centre has taken charge of every aspect of the problem – clinical, organizational and, if necessary, economic.

One of the proposals worthy of note at an international level is the creation of ‘crisis units’ which can carry out immediate investigation into the nature and cause of the adverse event [29]. This is a compulsory step in preparation for a meeting between heads of the crisis unit, the medical practitioners involved and the patients and their families, in which all the relevant information and explanations of the event and its causes are given, as well as proposals for economic compensation where deemed appropriate.

The clinical risk manager plays a key role in this scheme. He is reputedly skilled in communicating errors to patients and their families, in explaining prevention strategies adopted by the centre during the follow up stages with the patient and in arranging training courses in communication strategy with the health personnel [30]. 80% of the physicians who completed surveys believe there is a need for ad hoc training courses in error communication.

A recent study conducted by the University of Michigan and published in 2010 highlighted several economic reasons to justify a policy of error disclosure [31-33]. In a system which contemplates the possibility of immediate repair of injury suffered by a patient, it has been observed that the disclosure of error and adverse events does not lead to an increase in the number of lawsuits for compensation and would, in fact, help to reduce legal costs. After the implementation of an error disclosure programme, the average monthly number of applications for compensation fell from 7.03 to 4.52 per 100,000 patients, the monthly rate of appeals to a judicial tribunal fell from 2.13 to 0.7 per 100,000 patients and likewise, the average length of time needed to resolve medical disputes dropped from 1.36 to 0.95 years.

## Conclusions

Our study shows that Italian doctors without special training are not yet ready for the practice of error disclosure. The efficiency of a full disclosure policy on legal and administration costs is still far from being confirmed. Nevertheless, not enough studies have been carried out to be able to comprehend the economic impact of a full error disclosure policy, and so the debate continues.

## Additional file

Additional file 1:
**The questionnaire distributed directly to managerial members of the medical staff in the medical department, in surgery and in the service operating units of the IRCCS CROB.**
