# Supplementary material for: The right to be informed and fear of disclosure: sustainability of a full error disclosure policy at an Italian cancer centre/clinic
Source: BMC Health Serv Res. 2015 Apr 1;15:130. doi: 10.1186/s12913-015-0794-3 (PMC4460857; doi:10.1186/s12913-015-0794-3)
Supplement: Additional file 1: — The questionnaire distributed directly to managerial members of the medical staff in the medical department, in surgery and in the service operating units of the IRCCS CROB. [file 12913_2015_794_MOESM1_ESM.doc]

| **Additional file. Questionnaire.** | | | | | | | | | | | |  |  |  |  |  |
| --- | --- | --- | --- | --- | --- | --- | --- | --- | --- | --- | --- | --- | --- | --- | --- | --- |
| **Questions** | | |  |  |  | |  | | **Yes** | **No** | **Missing** | | | | | |
|  |  |  |  |  |  | |  | |  |  |  | | | | | |
| **Do you think that admitting a mistake to the patient is:** | | | | | | | |  | |  |  | | | | | |
| wrong |  |  |  |  |  |  | |  | |  |  | | | | | |
| an ethical and deontological duty | | | |  |  |  | |  | |  |  | | | | | |
| the patient's right | | |  |  |  |  | |  | |  |  | | | | | |
| necessary every time a mistake happens | | | | |  |  | |  | |  |  | | | | | |
| only necessary in cases of serious injury | | | | |  |  | |  | |  |  | | | | | |
| only necessary in cases of mild injury | | | |  |  |  | |  | |  |  | | | | | |
| only necessary in cases where the patient is not harmed | | | | | |  | |  | |  |  | | | | | |
| only necessary when the patient asks for an explanation | | | | | | | |  | |  |  | | | | | |
| helpful in avoiding medical lawsuits? | | | | | |  | |  | |  |  | | | | | |
|  |  |  |  |  |  |  | |  | |  |  | | | | | |
| **Do you think that admitting a mistake to the patient:** | | | | | | | |  | |  |  | | | | | |
| strengthens the patient’s trust in the doctor | | | | | |  | |  | |  |  | | | | | |
| reduces the risk of medical lawsuits | | | | |  |  | |  | |  |  | | | | | |
| reduces the probability of the same error being repeated | | | | | | | |  | |  |  | | | | | |
| reduces the patient's apprehensions about the likely outcome | | | | | |  | |  | |  |  | | | | | |
| reduces the probability that the patient will change doctors and/or hospital? | | | | | | | |  | |  |  | | | | | |
